# Biological sex and age influence GS-9620 activity ex vivo

**DOI:** 10.1172/jci.insight.182242

**Published:** 2025-05-06

**Authors:** Carissa S. Holmberg, Callie Levinger, Adam R. Ward, Alberto Bosque

**Affiliations:** 1 Department of Microbiology, Immunology, and Tropical Medicine, George Washington University, Washington, DC, USA.; 2 Division of Infectious Diseases, Department of Medicine, Weill Cornell Medicine, New York, New York, USA.6

**Keywords:** AIDS/HIV, Therapeutics, Innate immunity, Sex hormones

## Abstract

Toll-like receptors (TLRs) are being explored to enhance immunity in HIV cure strategies. The TLR7 agonist GS-9620 promotes immune activation, reactivates latent HIV, and delays viral rebound in some people with HIV. Previous work has shown that biological sex influences TLR7 signaling. This study examined the interplay between biological sex, age, and the sex hormones 17β-estradiol, progesterone, and testosterone on GS-9620’s ability to promote cytokine secretion and activate CD4^+^ T, CD8^+^ T, and NK cells ex vivo. Interestingly, sex hormones had no effect on GS-9620–mediated immune activation or cytokine induction. However, we found that GS-9620 activity was influenced by age only in female donors. Further, we found that GS-9620–mediated CD4^+^ T cell activation was positively correlated with the induction of IFN-γ and IL-12, while CD4^+^ T cell activation and IL-12 production were negatively correlated with age. Additionally, CD8^+^ T cell activation was positively correlated with IFN-γ production. Mechanistically, IFN-γ was sufficient to promote higher immune activation of both CD4^+^ and CD8^+^ T cells in female versus male donors. In conclusion, biological sex and age, but not sex hormones, influence GS-9620–mediated immune activation. Understanding these factors will help in designing and evaluating future clinical trials using GS-9620 for an HIV cure.

## Abstract 2

In this work we evaluated whether biological sex, age, and sex hormones influenced the activity of TLR7 agonist GS-9620 (Vesatolimod) for HIV cure approaches.

## Graphical abstract

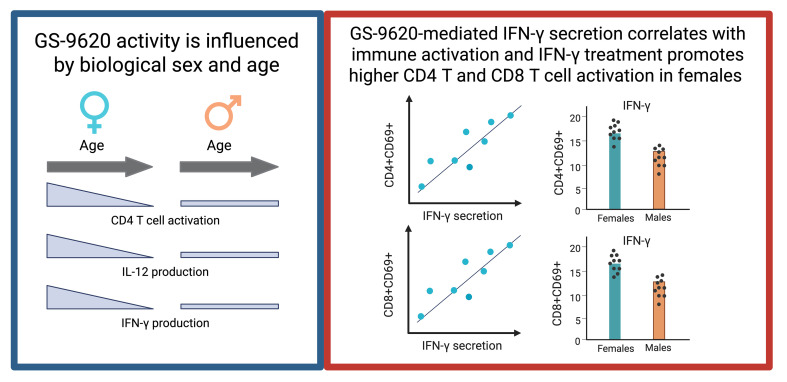

## Introduction

While HIV can be well managed with antiretroviral therapies (ARTs), treatment interruption leads to viral rebound within days to weeks in most people with HIV (PWH), as HIV persists in latent reservoirs (1–3). One proposed HIV cure strategy is termed “shock and kill,” in which latently infected cells are “shocked” with a latency-reversing agent (LRA) with subsequent “killing” of the now transcriptionally active cells by cytopathic effects from the virus or by the immune system (4). However, in addition to viral latency, another barrier to HIV cure is viral clearance by the immune system, which can be exhausted or dysfunctional because of chronic inflammation and persistent immune activation (5–8). Thus, an immune-enhancing agent, either the LRA itself or an agent given in combination, is likely needed to help clear the viral reservoir after HIV reactivation (9). One strategy of latency reversal and immune enhancement being investigated is the use of Toll-like receptor (TLR) agonists (10–12). Among them, TLR7 agonists have been shown to mediate both immune activation and reactivation of HIV. TLR7 recognizes single-stranded RNA (ssRNA) and is found primarily in the endosomal compartment of innate immune cells. Upon stimulation, TLR7 agonists induce a type I interferon (IFN) response, promoting not only the innate but also adaptive immune responses (13–15). In particular, the TLR7 agonist GS-9620 (clinically known as vesatolimod) has been well studied both as an LRA and as an immune-enhancing agent in vitro, ex vivo, and in vivo (16–22). GS-9620 has been shown to reactivate HIV from PBMCs ex vivo; however, this effect was not seen in isolated CD4^+^ T cells (16–18), possibly due to a lack of TLR7 expression on CD4^+^ T cells (11, 23). In vivo studies in rhesus macaques have shown that vesatolimod was well tolerated, upregulated interferon-stimulated genes (ISGs), increased IFN-α production, and activated CD4^+^ T, CD8^+^ T, and NK cells but had no significant effects on time to viral rebound or on the latent reservoir (19, 20). When vesatolimod treatment was combined with the broadly neutralizing antibody (bNAb) PGT121 in rhesus macaques, there were similar patterns of immune activation, but there was also delayed viral rebound in some macaques (21, 22). It is important to note that Borducchi et al. used the simian-human immunodeficiency virus (SHIV) viral strain SHIV162P3. This isolate displays a very high level of spontaneous control of viremia, even in the absence of ART, and ART was initiated early upon infection, limiting the potential clinical utility. Despite this, these studies highlight the potential therapeutic value of vesatolimod.

Recently, vesatolimod was tested in 2 clinical trials in PWH (24, 25). These clinical trials showed that vesatolimod was well tolerated in PWH on therapy (24, 25). Both studies showed increased immune activation, including of CD4^+^ T, CD8^+^ T, and NK cells; upregulated ISG expression; and increased cytokine production (24, 25). Interestingly, one of the clinical trials showed a modest decrease in intact proviral DNA while on ART and an increased time to rebound after ART interruption in HIV controllers (24), though the other study showed no significant difference in intact HIV DNA comparing vesatolimod groups with placebo (analytical treatment interruption was not conducted) (25). Together, the data suggest that vesatolimod/GS-9620 may not be sufficient alone to clear the viral reservoir or result in control of HIV upon therapy interruption but may be valuable as an immune-enhancing agent in conjunction with an LRA or other immunotherapies such as bNAbs.

It has been shown that TLR7 expression, which is encoded on the X chromosome, is influenced by biological sex (26–30). Specifically, TLR7 expression is higher in peripheral dendritic cells (pDCs), monocytes, and B cells from human females compared with males (27). This is also observed in pDCs from women living with HIV (31). Numerous studies have shown higher immune response to TLR7 stimulation in females, which is suggested to be caused by higher expression of TLR7 receptors but may also be influenced by regulation from 17β-estradiol (32–38). Because of this, it is important to point out that the abovementioned clinical trials with vesatolimod were conducted mostly in male participants, preventing analysis by biological sex, which may be a factor influencing the overall conclusions drawn from these studies. Further, some studies have also shown decreased TLR7 signaling activity with aging (16, 18, 24, 25, 39, 40). Overall, the potential biological impact of sex differences, sex hormones, and age are important factors to consider when designing immune-based HIV cure strategies, including interventions using vesatolimod. In this work, we evaluate ex vivo the role that biological sex, age, and sex hormones have on the activity of GS-9620.

## Results

### Age and sex hormones do not affect the ability of GS-9620 to induce IFN-α.

**Figure 1:**
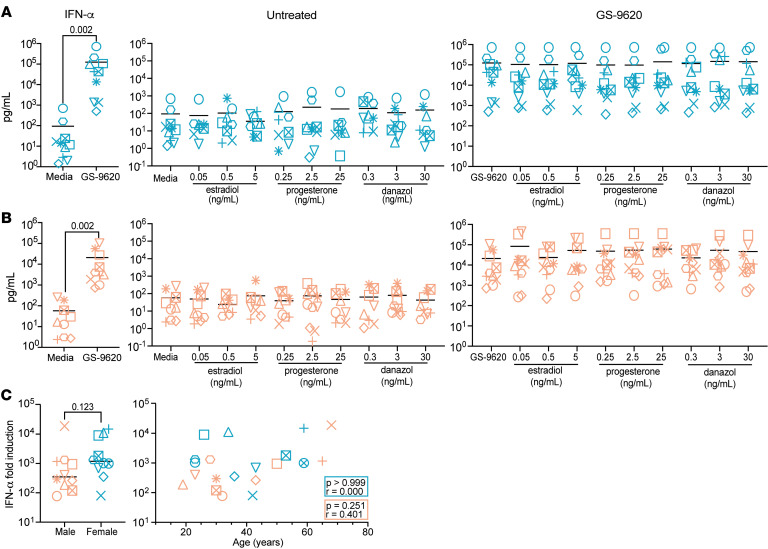
Age and sex hormones do not influence GS-9620–mediated production of IFN-α in PBMCs. Production of IFN-α in PBMCs from female donors (**A**) and male donors (**B**) with no stimulation compared with GS-9620 stimulation at 1 μM (first panel); pretreated with sex hormones estradiol (E2), progesterone (P4), and danazol (dana) at 3 concentrations (middle panel); and then GS-9620–stimulated with sex hormones E2, P4, and dana at 3 concentrations (last panel). (**C**) Comparative analysis of fold-induction of IFN-α production mediated by GS-9620 in male versus female donors (left) and fold-induction versus age (right). Teal symbols are female donors (*n* = 10), and peach symbols are male donors (*n* = 10). Multiple Wilcoxon’s tests, Mann-Whitney test, and nonparametric Spearman’s correlation were used to calculate *P* values.

TLR7 recognizes ssRNA and promotes the production of type I IFN, in particular IFN-α (41, 42). To understand the interplay between biological sex, age, and sex hormones on the activity of the TLR7 agonist GS-9620, we evaluated its ability to induce IFN-α in peripheral blood mononuclear cells (PBMCs) from 10 cisgender male and 11 cisgender female age-matched, HIV-seronegative donors (*P* = 0.641; mean ± SD male 38.8 ± 17.1 and female 42.3 ± 15.4) (Supplemental Table 1; supplemental material available online with this article; https://doi.org/10.1172/jci.insight.182242DS1). PBMCs were first pretreated overnight with physiological concentrations of 17β-estradiol (E2), progesterone (P4), or the testosterone derivative danazol. The pretreatment was done because sex hormones, such as E2, require at least 2 hours to induce genomic changes (43). Still in the presence of the sex hormones, cells were then stimulated with GS-9620 for 48 hours. Hormone concentrations used to treat the cells were determined based on the levels found in serum of cisgender females and males. To take into account shifts in hormone levels based on biological sex, age, and menstrual cycle, E2 concentrations of 0.05 ng/mL, 0.5 ng/mL, and 5 ng/mL were used to reflect concentrations found in cisgender males (between 0.01 and 0.082 ng/mL), during menstrual cycle shifts (up to 0.2 ng/mL during the follicular phase), and during pregnancy (up to 20 ng/mL in the third trimester) (44–47). For P4, concentrations of 0.25 ng/mL, 2.5 ng/mL, and 25 ng/mL were used to reflect levels in males (<0.20 ng/mL), during the luteal phase of the menstrual cycle (up to 25 ng/mL), and in menopause (< 0.4 ng/mL) (44, 48). For testosterone, we used a derivative danazol at 0.3 ng/mL, 3 ng/mL, and 30 ng/mL to reflect the levels of bioavailable testosterone found in younger male adults (3 ng/mL), older males (1.5 ng/mL), and females (0.2 ng/mL) (49–51). To avoid the presence of estrogenic compounds in fetal bovine serum (FBS) and with phenol red being a weak estrogen, we used media without phenol red and used charcoal-stripped serum (52, 53). After treatment, IFN-α2a (IFN-α) levels were measured in supernatants using an ultrasensitive Meso Scale Discovery (MSD) assay. IFN-α was induced in both male and female donors, but we did not observe an influence on IFN-α production from treatment with any of the sex hormones at any of the tested concentrations (Figure 1, A and B). In agreement with previous observations (31, 33, 34, 54), our data showed a trend toward higher induction of IFN-α in female donors compared with male donors, though it did not reach statistical significance (Figure 1C). Further analysis showed no correlation with the age of the donor (Figure 1C).

### Age negatively correlates with the ability of GS-9620 to promote immune activation.

**Figure 2:**
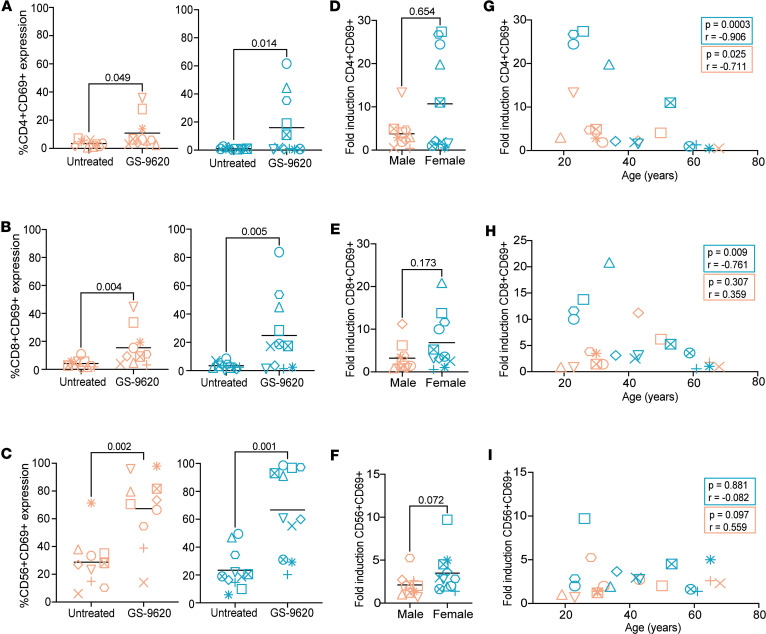
Age is negatively correlated with GS-9620–mediated activation of CD4^+^ T and CD8^+^ T cells. Activation of CD4^+^ T cells (**A**), CD8^+^ T cells (**B**), and NK cells (**C**) from male (left) and female (right) donors in no stimulation compared with GS-9620 stimulation at 1 μM. Comparative analysis of GS-9620–mediated fold-induction of CD69 on CD4^+^ T cells (**D**), CD8^+^ T cells (**E**), and NK cells (**F**) in male versus female donors. Analysis of GS-9620–mediated fold-induction of CD69 on CD4^+^ T cells (**G**), CD8^+^ T cells (**H**), and NK cells (**I**) versus age. Teal symbols are female donors (*n* = 11), and peach symbols are male donors (*n* = 10). Mann-Whitney tests and nonparametric Spearman’s correlations were used to calculate *P* values.

In addition to inducing IFN-α, GS-9620 has been shown to promote the activation of CD4^+^ T, CD8^+^ T, and NK cells, including HIV-specific responses (16, 18, 24, 25, 31). As such, we also evaluated the influence of biological sex, age, and sex hormones on the activation of these 3 cell types by GS-9620. As shown in Figure 2, A–C, GS-9620 induced activation measured as induction of the activation marker CD69 in CD4^+^ T, CD8^+^ T, and NK cells in both male and female donors (flow cytometry gating strategy shown in Supplemental Figure 1). We observed a trend toward higher fold-induction in immune activation of CD4^+^ T, CD8^+^ T, and NK cells in female donors compared with male donors. However, the response to GS-9620 between donors was highly variable, and this was not statistically significant (Figure 2, D–F). Immune activation was not influenced by the presence of any of the sex hormones (Supplemental Figure 2). Interestingly, GS-9620–mediated immune activation of CD4^+^ T cells in females was strongly negatively correlated with age (*r* = 0.906, *P* < 0.001), with younger donors showing higher fold immune activation than older donors (Figure 2G). This negative correlation was also observed in male donors, but the levels of induction were in general lower (mean 3-fold) compared with female donors (mean 10-fold). This negative correlation was also observed for CD8^+^ T cell activation but only in the female donors (Figure 2H). On the other hand, GS-9620–mediated NK cell activation in female and male donors was not influenced by age (Figure 2I). Overall, GS-9620–mediated activation was seen in all 3 cell types, with generally higher induced activation in female donors. Additionally, age negatively influenced the ability of GS-9620 to promote CD4^+^ and CD8^+^ T cell activation.

### Correlates of GS-9620–mediated CD4^+^ and CD8^+^ T cell immune activation.

**Figure 3:**
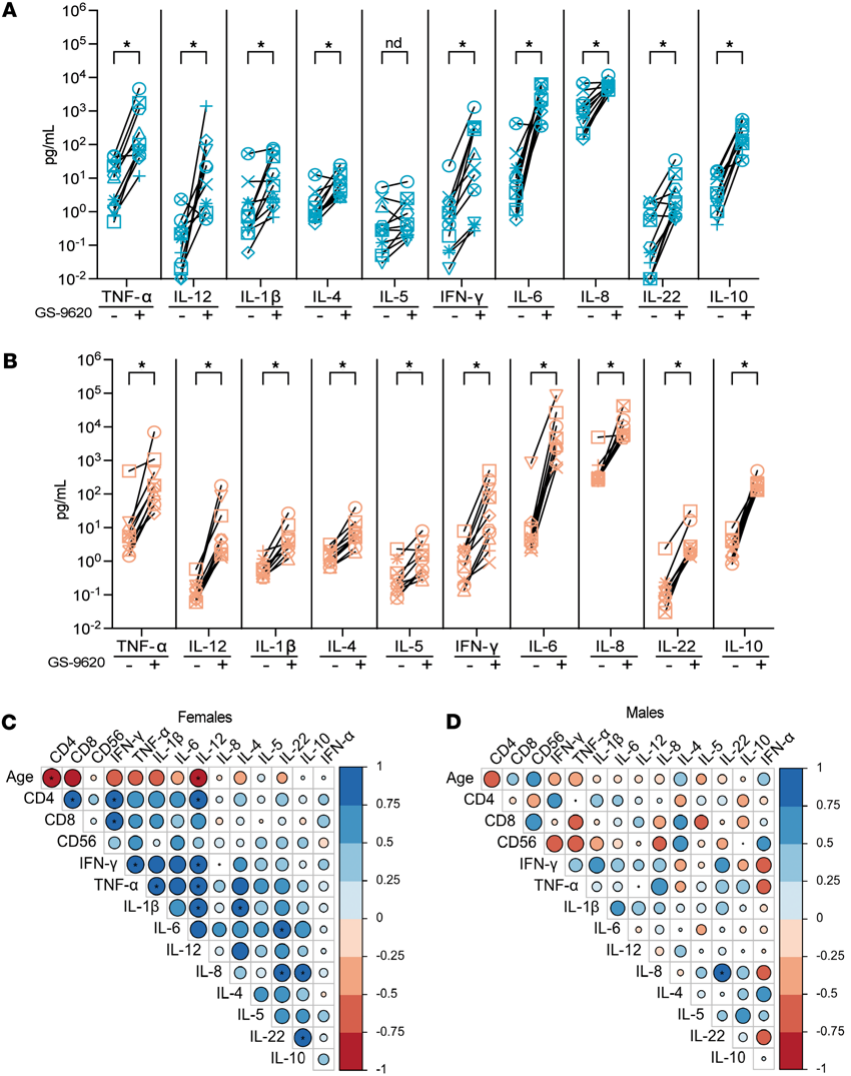
GS-9620–mediated production of 10 cytokines and correlation matrix with immune cell activation and cytokine production. Levels of each indicated cytokine from unstimulated (–) or GS-9620–stimulated (+) PBMCs in females (**A**) and males (**B**). Teal symbols are female donors (*n* = 11), and peach symbols are male donors (*n* = 10). Multiple Wilcoxon tests were used to calculate significance with multiple comparison using the method of Benjamini, Krieger, and Yekutieli. nd, no statistically significant difference. Multiple comparison correlation matrix of age and GS-9620–mediated CD4^+^ T cell activation, CD8^+^ T cell activation, NK cell activation, and production of IFN-γ, IL-1β, IL-4, IL-5, IL-6, IL-8, IL-10, IL-12p70, IL-22, TNF-α, and IFN-α in females (**C**) and males (**D**) showing significance (*q < 0.05) and *r* value by size and shade of circle and positive (blue) or negative (red) correlation by color. The *P* values were adjusted using the FDR method of Benjamini and Hochberg.

**Figure 4:**
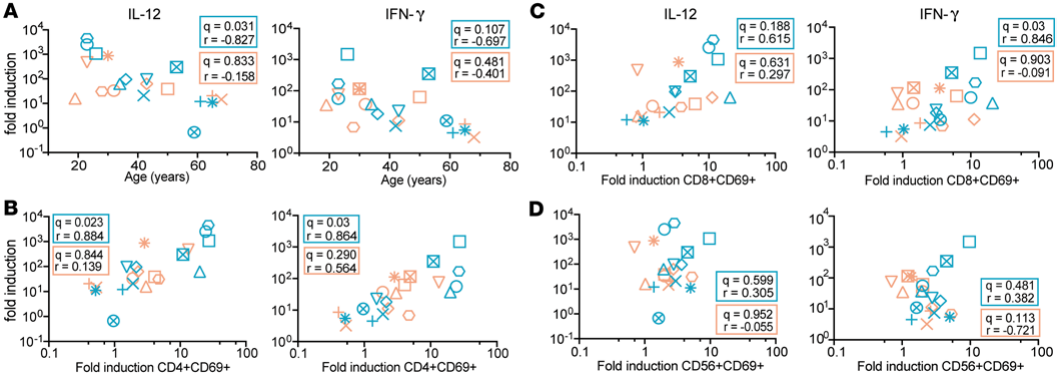
GS-9620–mediated fold-induction of IL-12 and IFN-γ is correlated with age and immune cell activation in female donors. Fold-induction of IL-12 (left) and IFN-γ (right) versus age (**A**), CD4^+^ T cells (**B**), CD8^+^ T cells (**C**), and NK cells (**D**). Teal symbols are female donors (*n* = 11), and peach symbols are male donors (*n* = 10). The *P* values were adjusted using the FDR method of Benjamini and Hochberg.

There are conflicting studies on the expression of TLR7 receptors on CD4^+^ and CD8^+^ T cells, though it is well established that TLR7 receptors are expressed on dendritic cells, NK cells, and B cells (55, 56). In consideration of this, the immune activation mediated by GS-9620 is thought to be an indirect effect (57–59). Previous work by Tsai et al. demonstrated that IFN-α was involved in the immune activation of CD4^+^ and CD8^+^ T cells mediated by GS-9620 (18). Since we observed an age-dependent effect on immune activation in female donors but did not observe the same for IFN-α production, we hypothesized that other cytokines may contribute to this immune activation. Along with type I IFN, pro-inflammatory cytokines such as IFN-γ, IL-1β, IL-12, IL-6, and TNF-α are induced upon TLR7 signaling (15, 60). Therefore, using the same supernatants that we measured IFN-α from, we measured the ability of GS-9620 to promote the induction of a panel of 10 cytokines that included IFN-γ, IL-1β, IL-4, IL-5, IL-6, IL-8, IL-10, IL-12p70, IL-22, and TNF-α. All 10 cytokines were induced with GS-9620 treatment in both male and female donors (Figure 3, A and B). As we observed with IFN-α and immune cell activation, we did not see any significant influence from sex hormones on cytokine induction (Supplemental Figures 3–12). To identify correlates associated with GS-9620–mediated immune cell activation, we performed correlation analysis between age; fold-induction of CD69 on CD4^+^ T, CD8^+^ T, and NK cell activation; and fold-induction of IFN-α and the 10 cytokines in both female and male donors, adjusted for multiple comparisons (Figure 3, C and D). Interestingly, we observed a negative correlation with age and IL-12 induction only in the female donors (Figure 3, C and D, and Figure 4A). On the other hand, there was a strong positive correlation between CD4^+^ T cell activation and IL-12 and IFN-γ induction in female donors but not in male donors (Figure 3, C and D, and Figure 4B). Additionally, induction of IFN-γ was also positively correlated with CD8^+^ T cell activation but again only in female donors (Figure 3, C and D, and Figure 4C). We observed no correlation between NK cell activation and IFN-γ or IL-12 induction (Figure 4D). In summary, GS-9620–mediated immune activation of CD4^+^ and CD8^+^ T cells correlated with the induction of pro-inflammatory cytokines IL-12 and IFN-γ, but not IFN-α, only in female donors.

### Higher IFN-γ–mediated CD4^+^ and CD8^+^ T cell immune activation in female donors.

**Figure 5:**
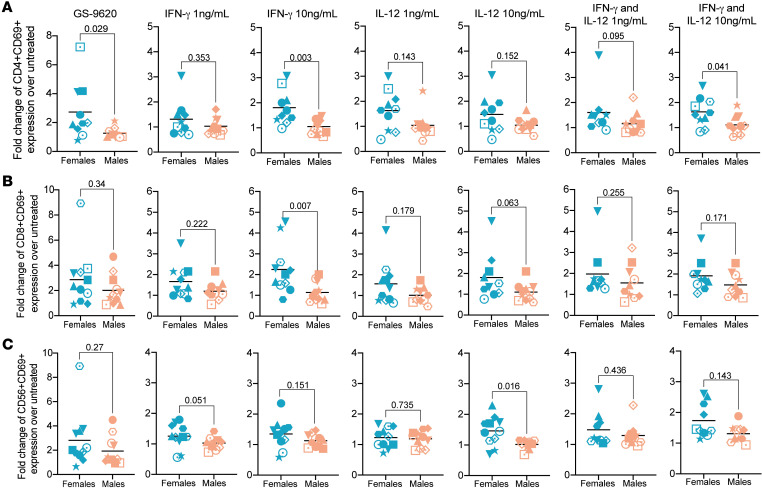
Fold-induction of IFN-γ–treated cells shows higher immune cell activation in female donors. CD69 fold-induction on CD4^+^ T cells (**A**), CD8^+^ T cells (**B**), and NK cells (**C**) treated with GS-9620 (first panel), IFN-γ at 1 and 10 ng/mL (second and third panels), IL-12 at 1 and 10 ng/mL (fourth and fifth panels), and a combination of both IFN-γ and IL-12 at 1 and 10 ng/mL (sixth and seventh panels). Teal symbols are female donors (*n* = 11), and peach symbols are male donors (*n* = 11). Mann-Whitney tests were used to calculate *P* values.

As we observed positive correlations between GS-9620–mediated immune activation of CD4^+^ and CD8^+^ T cells and the secretion of pro-inflammatory cytokines IFN-γ and IL-12, we wanted to verify whether the activity of either of these cytokines could explain the higher immune activation mediated by GS-9620 in female compared with male donors. PBMCs were treated with 1 ng/mL or 10 ng/mL of IFN-γ or IL-12 either alone or in combination. Similar to what we observed with GS-9620–mediated activation (Figure 5A), CD4^+^ T cells treated with 10 ng/mL IFN-γ showed higher activation in female donors compared with male donors either alone or in combination with IL-12 (Figure 5A). This was also true in CD8^+^ T cells treated with 10 ng/mL IFN-γ, though the combination with IL-12 was not statistically significant (Figure 5B). Further, we observed a statistically significant sex difference in NK cell activation only by treatment with 10 ng/mL IL-12 but not with IFN-γ alone or the combination (Figure 5C). Interestingly, we observed no correlation in female donors between age and CD4^+^ or CD8^+^ T cell activation mediated by IL-12 either alone or in combination with IFN-γ (Supplemental Figure 13). This suggests that the negative correlation observed with GS-9620–mediated CD4^+^ T cell activation with age may not be associated with different ability of cytokines to promote immune activation in PBMCs from older female donors but rather with a lower secretion of different cytokines upon GS-9620 stimulation associated with aging.

## Discussion

In this study, we investigated the potential impacts of biological sex, age, and sex hormones on the immune activation properties of GS-9620. We observed higher activity of GS-9620 in female donors compared with male donors. This is in agreement with previous studies showing a sex-specific bias in females in response to TLR7 signaling, including an X-chromosome bias, which has been shown to result in higher interferon regulatory factor 5 (IRF5) expression and higher IFN-α production (32, 35, 36, 38). Interestingly, we observed a strong negative correlation between age and GS-9620 immune activation. Though an association with age and decreased TLR7 signaling activity has been shown previously (16, 18, 24, 25, 39, 40), our study observed that this correlation was stronger in female donors. To our knowledge, this is the first time an association between GS-9620 activity and age specifically in female, but not male, donors has been reported. A study by Sridharan et al. suggests that a possible mechanism for this difference is impaired pDC response to TLR7 signaling with aging, resulting in an inhibition of the ability of TLR7 agonists to induce CD4^+^ and CD8^+^ T cell proliferation and IFN-γ secretion (61). While that study did not differentiate response by sex, it did include over 50% female donors in both the young and aging populations. Our study suggests that younger female donors may be more responsive to TLR7 agonists, with CD4^+^ and CD8^+^ T cells showing less activation with older age. This is an important consideration when using TLR7 agonists for HIV cure strategies, as much of the current population of PWH in the United States is aging (62). In sub-Saharan Africa, however, young women and girls are disproportionately affected, with 77% of new HIV infections being among 15- to 24-year-olds (63, 64). This demographic could benefit from cure strategies that are more effective in young female populations, such as TLR7 agonists. These are important considerations for the design of future clinical trials for HIV cure.

Previous studies have implicated IFN-α as the cytokine responsible for TLR7-mediated immune activation; however, we did not observe a correlation between IFN-α induction and age that could explain our results, though this may be partially due to small sample size (18). Since we did not observe this, we conducted further analysis on other pro-inflammatory cytokines, which identified IL-12 and IFN-γ as potential cytokines associated with GS-9620–mediated activation of both CD4^+^ and CD8^+^ T cells in female donors. More specifically, we observed an age-dependent induction of both cytokines only in female donors, with younger female donors inducing higher levels of both cytokines. Both IL-12 and IFN-γ, which are induced by pDCs, promote CD4^+^ and CD8^+^ T cell activation (65, 66). Therefore, impaired pDC function could be affecting CD4^+^ and CD8^+^ T cell activation and secretion of other cytokines in older donors (61). This is also supported by the fact that we did not see a correlation between activation of T cells and age in either females or males when cells were treated with IL-12 and IFN-γ directly, meaning it is likely not due to the direct activation of CD4^+^ and CD8^+^ T cells mediated by the cytokines but a lesser induction of the cytokines associated with aging. Further, our study saw a trend of higher NK cell activation in female donors compared with male donors. A previous study has shown that NK cells can be directly activated by TLR7 agonists as well as by IL-12 and IFN-α (56). Since females have been shown to have higher TLR7 expression, this may be the reason for the trend in higher NK cell activation in female donors.

We observed no effects from physiologically relevant levels of sex hormones on GS-9620–mediated immune activation. Studies in mice have shown modulation of TLR7 signaling in pDCs by E2; however, this has not been confirmed in humans (36–38). These studies showed that E2 enhanced TLR7 signaling and that ablation of estrogen receptor-1 in mouse hematopoietic cells reduced *IRF5* mRNA expression in pDCs and reduced IFN-α production. Unlike these studies, we did not directly measure pDC activity in isolation and instead tested for effects of sex hormones on activation of PBMCs. There may also be limited effects of studying E2 in vitro as it is a short exposure time. It is worth addressing that it is difficult to model the complex nature of the interplay between sex hormones and the immune system in vivo in an ex vivo setting.

Previous studies of TLR7 activation in the context of HIV cure treatments have also shown differences by biological sex (54, 67). These previous works have established higher production of IFN-α from female participants (54, 67). Further, Chang et al. found that ISG expression was higher in treatment-naive females with chronic HIV than in males, which was associated with higher T cell immune activation (67). Meier et al. also found a trend toward lower IFN-α levels in postmenopausal women in response to a TLR7 HIV-1–derived ligand, further suggesting possible effects from sex hormones. Additionally, the impact of TLR7 stimulation on HIV viral reservoir size has been investigated, with an inverse correlation seen between intact proviral HIV DNA levels and IFN-α and TNF-α responses of pDCs in a cohort of cisgender, ART-suppressed women (68). Importantly, previous work in our lab showed essentially no difference in response to GS-9620 in HIV-negative donors compared to PWH (16). Overall, these studies show how sex differences may influence HIV cure strategies.

One limitation to our study is that we did not directly measure pDC activity. This may account for why we only saw a trend of increased IFN-α in female donors over male donors (33, 34, 54). There are contrary studies on whether IFN-α production is associated with aging. Our study did not show differences in levels of IFN-α induced by TLR7 stimulation in older versus younger participants, but some studies have shown either higher or lower production of IFN-α in older populations (40, 61, 69). This may be due to measuring IFN-α production in PBMCs as opposed to isolated pDCs or by differing methods by which each of these studies measured IFN-α production. For example, Splunter et al. (69) and Panda et al. (40) measured IFN-α production intercellularly in pDCs. There is also a study showing that polymorphisms in TLR7 expression greatly affect activation and thus IFN-α production by individuals (70), which was seen at a similar rate in women living with HIV (31). Another limitation is our small sample size; we had 4 female donors over the age of 50, thus limiting our power in measuring pre- versus postmenopausal activation and the impacts of age across the adult lifespan.

In conclusion, our study highlights the interplay between age and biological sex influencing the activity of GS-9620. Several mechanisms could be involved in differences seen in GS-9620–mediated activation, including direct versus indirect stimulation of different cell populations, IFN-α regulation of cytokines, or regulation by other cytokines. Overall, this study indicates the need for future studies to investigate mechanisms of sex differences and aging in TLR7 signaling in the context of HIV cure strategies. Based on our ex vivo studies, future clinical trials should also measure outcomes considering age and biological sex of the participants involved in HIV cure strategy studies using TLR7 agonists.

## Methods

### Sex as a biological variable.

All experiments used an equal number of male and female donors to examine differences by biological sex.

### Reagents.

GS-9620 and sex hormones were obtained from Cayman Chemical Company: GS-9620 (catalog 19628), 17β-estradiol (catalog 10006315), progesterone (catalog 15876), and danazol (catalog 16471). IFN-γ (catalog 506532) was obtained from BioLegend, and IL-12 (catalog 200-12) was obtained from PeproTech.

### PBMCs.

Buffy coats were obtained from HIV-negative donors aged 17 years and older from the Gulf Coast Regional Blood Center, or frozen PBMCs from STEMCELL Technologies were used (catalog 70025). PBMCs were isolated from buffy coats by Lymphoprep cell gradient centrifugation (STEMCELL Technologies, catalog 07851). After washing 2 times in PBS + EDTA (2 mM), the PBMCs were resuspended in RPMI 1640 medium (R&D Systems, Bio-Techne, catalog M30450) supplemented with 10% charcoal-stripped human FBS (Gibco, Thermo Fisher Scientific), 1% l-glutamine, and 1% penicillin/streptomycin (Gibco, Thermo Fisher Scientific).

### Immune activation.

Briefly, PBMCs from 10 cisgender male and 11 cisgender female HIV-seronegative donors were cultured at 3 × 10^6^/mL overnight in RPMI without phenol red and charcoal-stripped serum with treatment of 17β-estradiol (0.05, 0.5, and 5 ng/mL), progesterone (0.25, 2.5, 25 ng/mL), or danazol (0.3, 3, 30 ng/mL). For experiments without hormone treatment, PBMCs were rested overnight without treatment. Then after overnight incubation, GS-9620 was added at 1 μM, or IL-12 and/or IFN-γ was added at 1 or 10 ng/mL for an additional 48 hours. Cells were collected at 1 × 10^6^/mL and stained for flow cytometry analysis with CD3, CD4, CD8, CD56, and CD69 markers, and supernatants were frozen at –20°C for cytokine analysis, outlined below.

### Cytokine and IFN-α analysis.

Frozen supernatants were thawed, and the assay was performed according to Quanterix manufacturer protocol. Briefly, samples were incubated on the 96-well, pre-spotted plate for 2 hours at room temperature (RT) with shaking at 525 rpm. The plate was washed with Quanterix wash buffer (catalog 1863332) using a plate washing and then incubated first with biotinylated antibody and then with streptavidin-HRP (both from the kit) for 30 minutes each at RT and shaking at 525 rpm. Plates were washed between each step. After final incubation, the plate was washed with a 5-step wash, kit SuperSignal was added, and the plate was read. Ten cytokines were measured using Quanterix SP-X Corplex Cytokine Panel (IFN-γ, IL-1β, IL-4, IL-5, IL-6, IL-8, IL-10, IL-12P70, IL-22, TNF-α) (product 85-0329). IFN-α was measured from the same supernatants using MSD Human IFN-α2a Ultra-Sensitive Kit (catalog K151P3S-1)

### Flow cytometry.

With immune activation antibodies, 1 × 10^6^ cells were washed with PBS + 3% FBS, then incubated with human Fc block for 10 minutes (catalog 564220, BD Biosciences), and then cells were stained with surface markers, anti-CD3 BV786 (clone SP34-2, catalog 563800, BD Biosciences), anti-CD4 FITC (clone RPA-T4, catalog 300538, BioLegend), anti-CD8 PE (clone OKT-8, Thermo Fisher Scientific, catalog 12-0086-42), anti-CD56 PerCP/Cy5.5 (clone 5.1H11, BioLegend, catalog 362506), anti-CD69 APC-Cy7 (clone FN50, BioLegend, catalog 310914), and eBioscience Fixable Viability Dye eFluor 450 (Thermo Fisher Scientific, catalog 65-0863-18), for 30 minutes at 4°C.

All experiments were run on a BD LSR Fortessa X20 flow cytometer with FACSDiva software or on an Aurora Cell Analyzer (Cytek Biosciences). Data were analyzed using FlowJo (TreeStar, Inc.).

### Statistics.

The following statistical analyses were performed using GraphPad Prism 9.4.1 software: Wilcoxon’s paired, nonparametric (comparing agonist treated and untreated); Kruskal-Wallis (comparing hormone treatment groups); Mann-Whitney *U* unpaired, nonparametric test (comparing biological sex); and Spearman’s correlation tests (age analysis). Figure 3B and Figure 4 were analyzed using a MULTTEST procedure in SAS, adjusting the *P* values using the FDR method of Benjamini and Hochberg. A *P* value less 0.05 was considered significant. All the data with error bars are presented as mean values ± SD. The statistical analysis used is indicated in each figure legend.

### Study approval.

Volunteers 17 years and older at the Gulf Coast Regional Blood Center served as blood participants. WBC concentrates (buffy coat) prepared from a single unit of whole blood by centrifugation were purchased from STEMCELL Technologies.

### Data availability.

All data are available in the main text, the supplement, or the Supporting Data Values XLS file.

## Author contributions

Work was conceptualized by AB and CSH. CSH, CL, and AB developed the methodology. Investigation was done by CSH and CL. Visualization was done by CSH, ARW, and AB. Funding acquisition was done by AB and CSH. Project administration was done by AB. Supervision by AB. CSH and AB wrote the original manuscript draft. CSH, ARW, and AB reviewed and edited the manuscript. All the authors approved the final version.

## Supplementary Material

Supplemental data

Supporting data values

## Version 1 05/06/2025

In-Press Preview

## Version 2 06/23/2025

Electronic publication
