# Supplementary material for: Biological sex and age influence GS-9620 activity ex vivo
Source: JCI Insight. 2025 May 6;10(12):e182242. doi: 10.1172/jci.insight.182242 (PMC12220944; doi:10.1172/jci.insight.182242)
Supplement: Supplemental data [file jciinsight-10-182242-s016.pdf]

Supplementary Materials for

Biological sex and age influence GS-9620 activity *ex vivo*

Carissa S. Holmberg, Callie Levinger, Adam Ward, and Alberto Bosque

Corresponding author: abosque@gwu.edu

The PDF file includes:

Table 1

Figures S1 to S13

Donor ID	Symbol	Source	Biological sex	Age	Race/ethnicity	Height (cm)	Weight (kg)	Blood type	Smoker status	BMI
D001	Circle	STEMCELL	Female	17	Mixed	166	84	O+	No	30.5
D002	Asterisk	STEMCELL	Male	30	Caucasian	170	80	A+	No	27.7
D003	Circle	STEMCELL	Male	32	Caucasian	177	82	A+	yes	26.2
D004	Square	STEMCELL	Female	26	Asian	159	58	AB+	No	22.9
D005	Diamond	STEMCELL	Female	36	Caucasian	165	92	O+	no	33.8
D006	Square	STEMCELL	Male	50	Caucasian	173	116	A+	yes	38.8
D007	Diamond	STEMCELL	Male	43	Mixed	183	110	O+	no	32.8
D008	Hexagon	STEMCELL	Female	23	Mixed	166	84	O+	no	30.5
D009	Hexagon	STEMCELL	Male	28	Asian	169	75	O+	no	26.3
D010	Triangle	STEMCELL	Female	34	Caucasian	157	95	O-	no	38.5
D011	Triangle	STEMCELL	Male	23	Caucasian	198	152	A+	yes	38.8
D012	Inverted Triangle	STEMCELL	Female	43	Caucasian	185	76	B-	yes	22.2
D013	Inverted Triangle	STEMCELL	Male	19	Caucasian	179	75	O+	yes	23.4
D014	Square with X	STEMCELL	Female	53	Caucasian	170	91	O+	no	31.5
D015	Square with X	STEMCELL	Male	30	Asian	180	79	O+	no	24.4
D016	Cross	Gulf Coast Blood Bank	Female	65	N/A	N/A	N/A	O+	N/A	N/A
D017	X	Gulf Coast Blood Bank	Female	61	N/A	N/A	N/A	O+	N/A	N/A
D018	Asterisk	Gulf Coast Blood Bank	Female	42	N/A	N/A	N/A	B+	N/A	N/A
D019	X	Gulf Coast Blood Bank	Male	68	N/A	N/A	N/A	O+	N/A	N/A
D020	Cross	Gulf Coast Blood Bank	Male	65	N/A	N/A	N/A	A+	N/A	N/A
D021	Circle with x	Gulf Coast Blood Bank	Female	58.9	N/A	N/A	N/A	A+	N/A	N/A

Table 1. Table of donors for GS-9620-mediated immune activation.

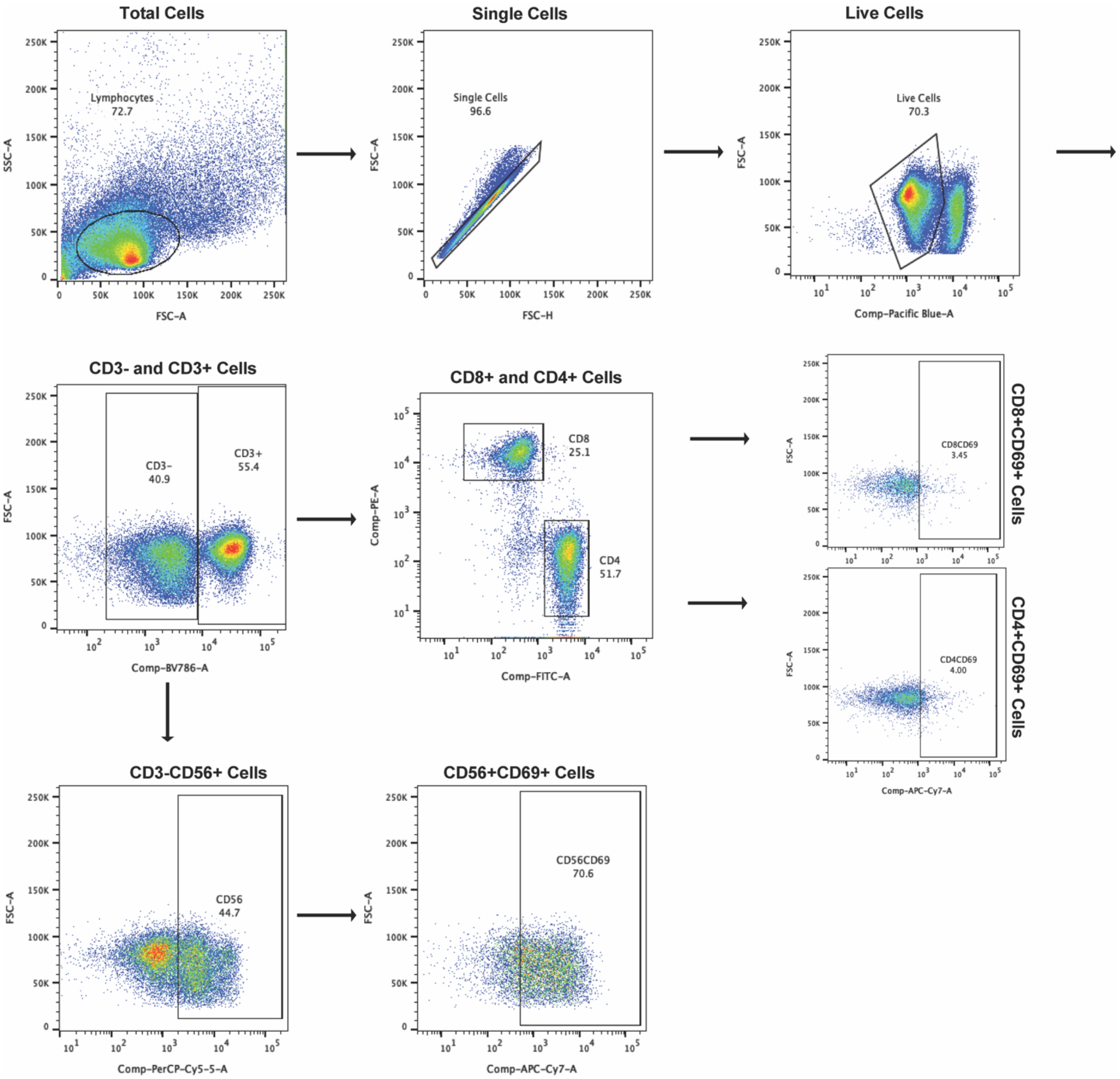

SF1. Representative flow plot of PBMCs.

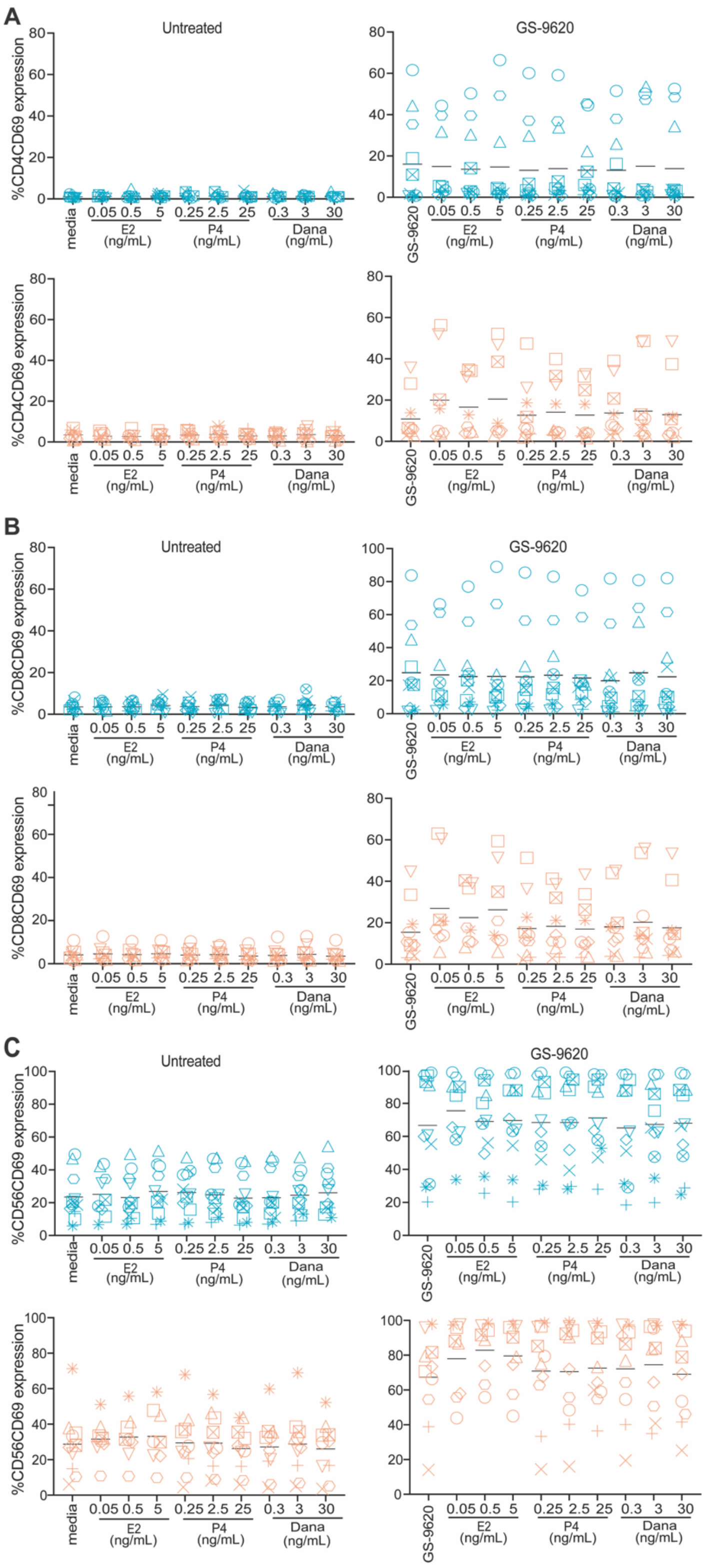

SF2. Analysis of GS-9620-mediated CD4 T cells, CD8 T cells, and CD56 cell activation in PBMCs in the presence of sex hormones. %CD4CD69 positive cells in untreated and hormone treatments of estradiol (E2), progesterone (P4), and danazol (dana) alone (left panel) or in the presence of GS-9620 (right panel), %CD8CD69 positive cells (B), and %CD56CD69 positive cells (C). Teal symbols are female donors, and peach symbols are male donors.

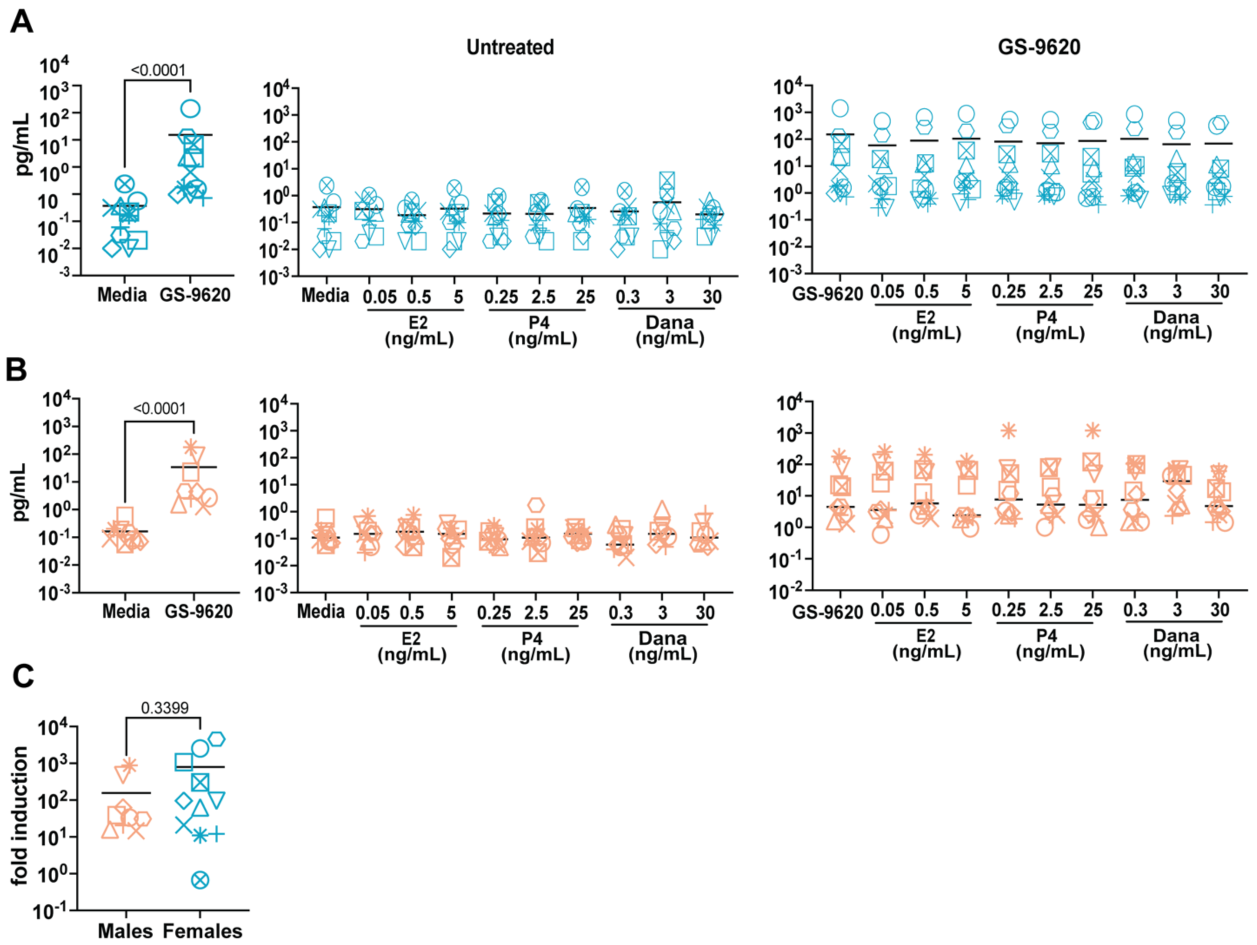

SF3. Analysis of GS-9620-mediated IL-12 induction in PBMCs in the presence of sex hormones. IL-12 production before and after stimulation with GS-9620 (first panel), hormone treatments of estradiol (E2), progesterone (P4), and danazol (dana) alone (middle panel), or in the presence of GS-9620 (right panel). Teal symbols are female donors (A), and peach symbols are male donors (B). (C) Fold induction of IL-12 production by biological sex. Mann-Whitney test was used to calculate p values.

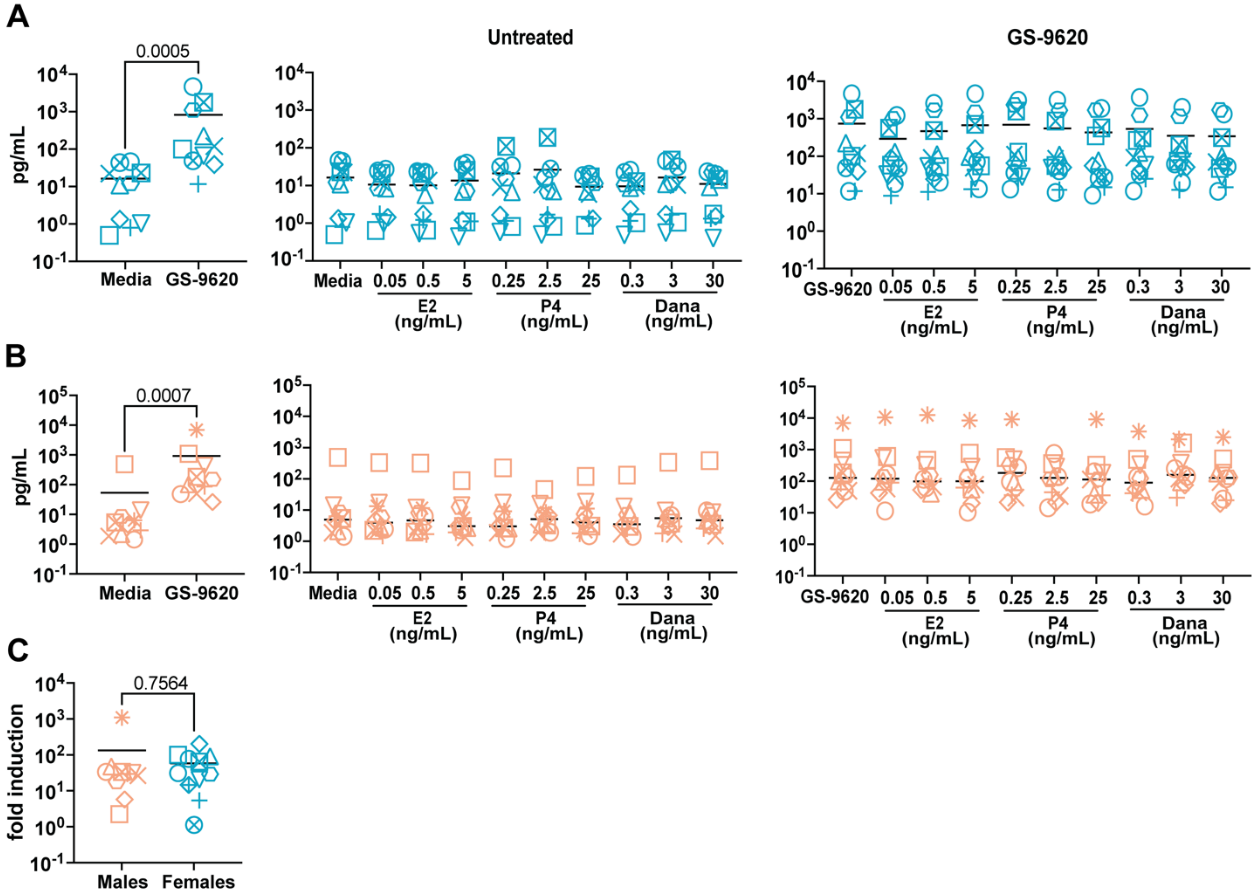

SF4. Analysis of GS-9620-mediated TNF-α induction in PBMCs in the presence of sex hormones. TNF-α production before and after stimulation with GS-9620 (first panel), hormone treatments of estradiol (E2), progesterone (P4), and danazol (dana) alone (middle panel), or in the presence of GS-9620 (right panel). Teal symbols are female donors (A), and peach symbols are male donors (B). (C) Fold induction of TNF-α production by biological sex. Mann-Whitney test was used to calculate p values.

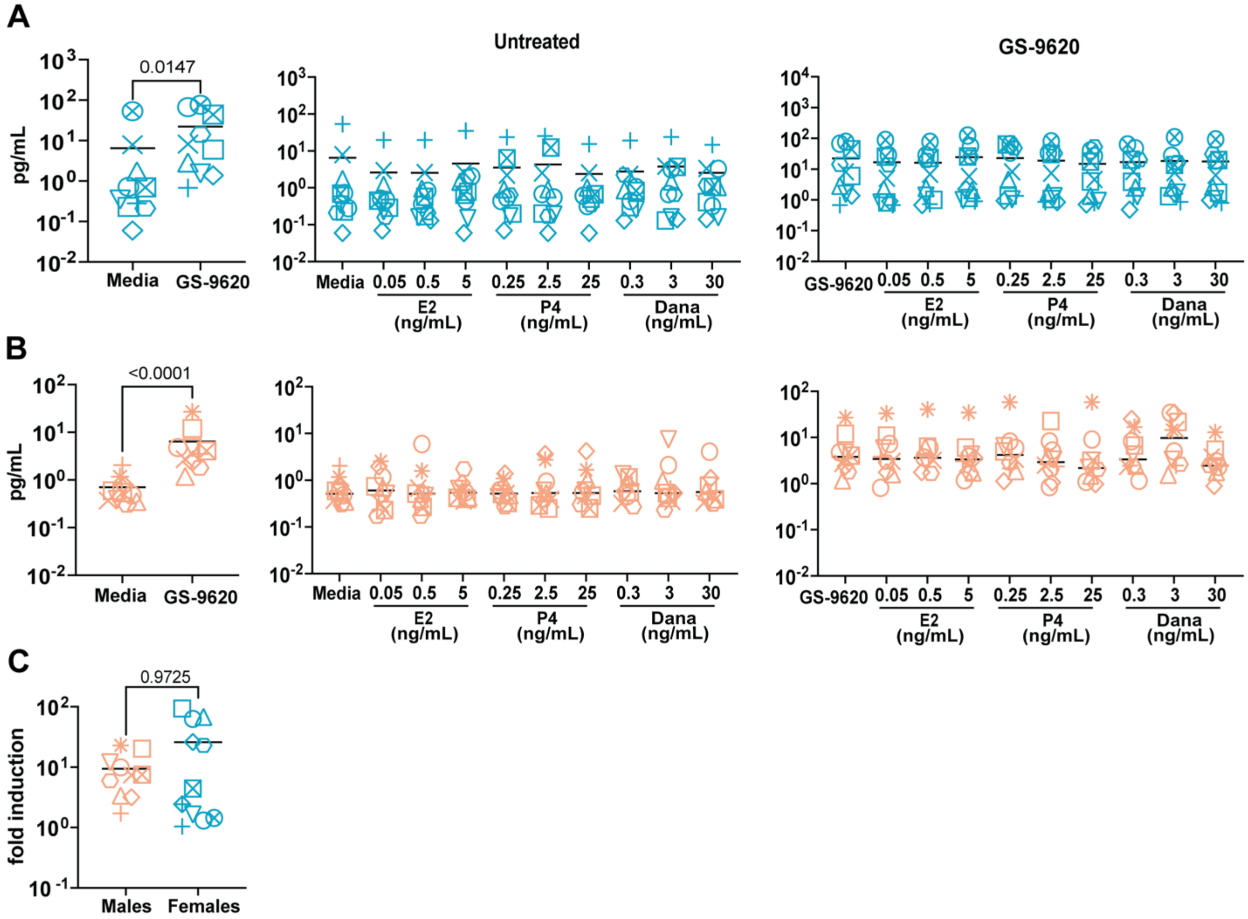

SF5. Analysis of GS-9620-mediated IL-1β induction in PBMCs in the presence of sex hormones. IL-1β production before and after stimulation with GS-9620 (first panel), hormone treatments of estradiol (E2), progesterone (P4), and danazol (dana) alone (middle panel), or in the presence of GS-9620 (right panel). Teal symbols are female donors (A), and peach symbols are male donors (B). (C) Fold induction of IL-1β production by biological sex. Mann-Whitney test was used to calculate p values.

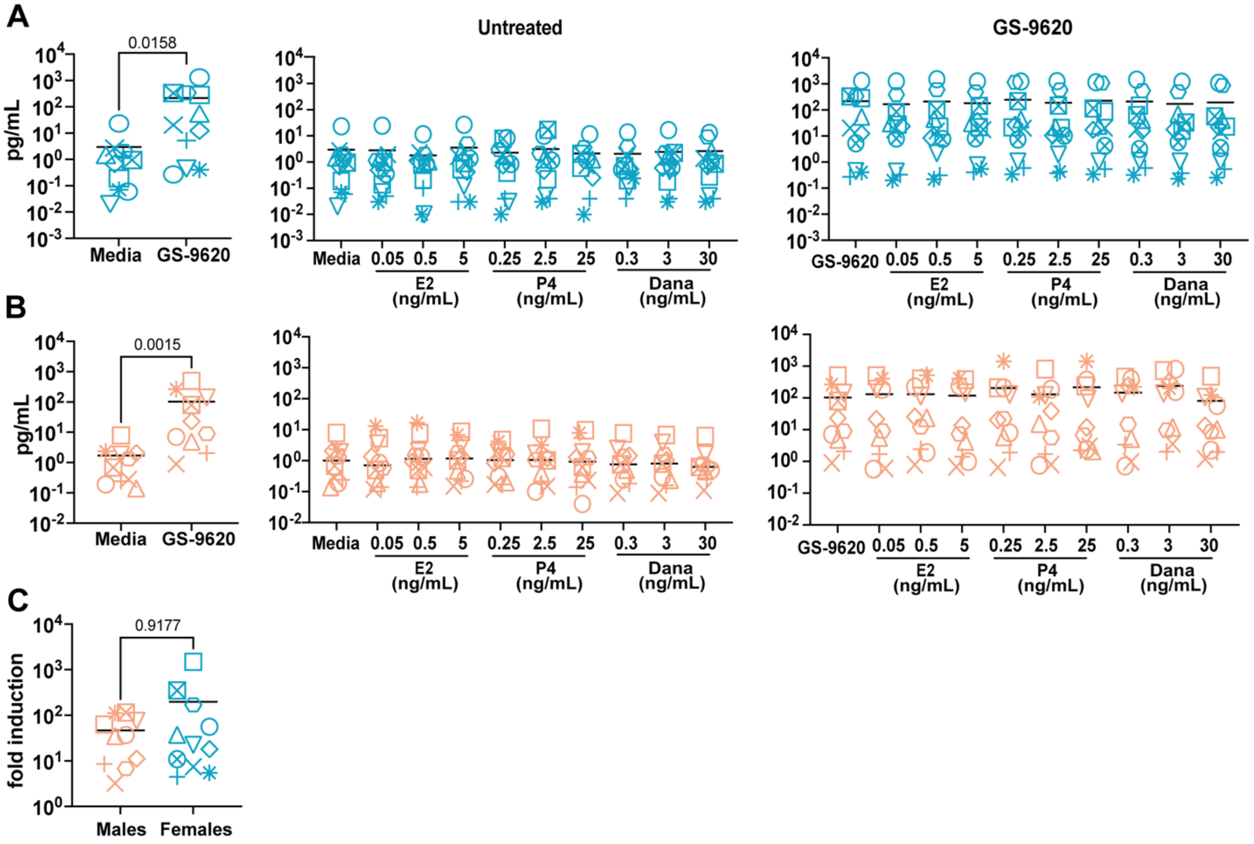

SF6. Analysis of GS-9620-mediated IFN-γ induction in PBMCs in the presence of sex hormones. IFN-γ production before and after stimulation with GS-9620 (first panel), hormone treatments of estradiol (E2), progesterone (P4), and danazol (dana) alone (middle panel), or in the presence of GS-9620 (right panel). Teal symbols are female donors (A), and peach symbols are male donors (B). (C) Fold induction of IFN-γ production by biological sex. Mann-Whitney test was used to calculate p values.

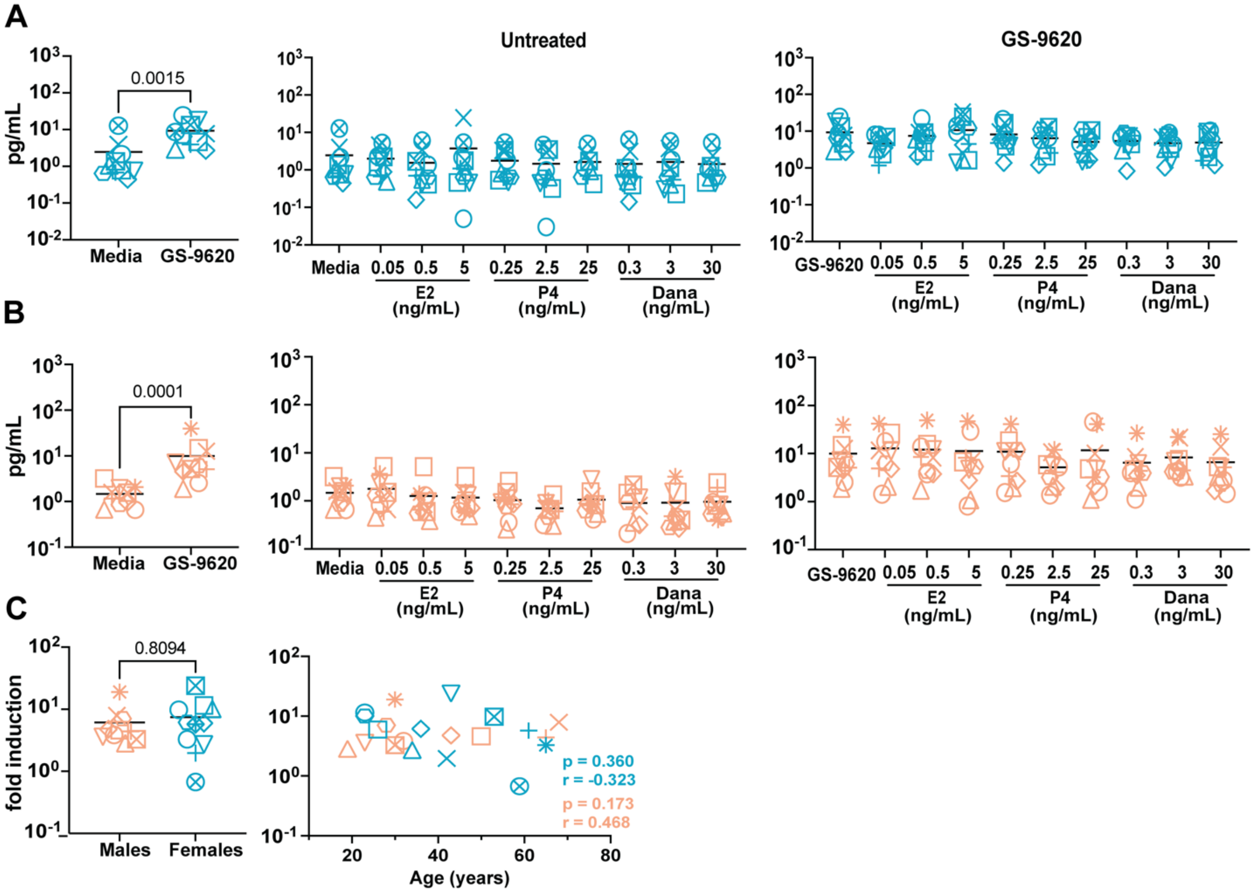

SF7. Analysis of GS-9620-mediated IL-4 induction in PBMCs in the presence of sex hormones. IL-4 production before and after stimulation with GS-9620 (first panel), hormone treatments of estradiol (E2), progesterone (P4), and danazol (dana) alone (middle panel), or in the presence of GS-9620 (right panel). Teal symbols are female donors (A), and peach symbols are male donors (B). (C) Fold induction of IL-4 production by biological sex (left) and age (right). Mann-Whitney test and nonparametric spearman correlation were used to calculate p values.

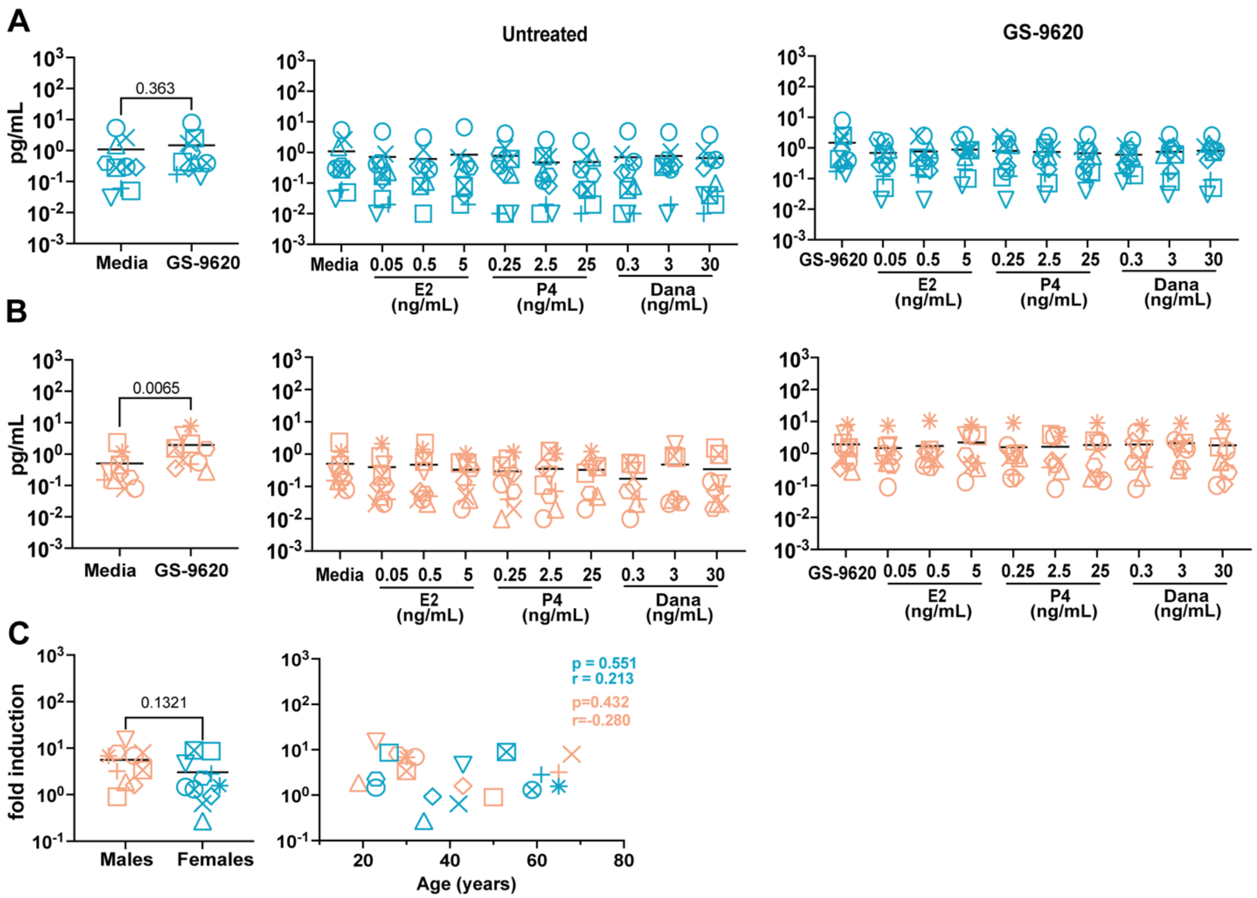

SF8. Analysis of GS-9620-mediated IL-5 induction in PBMCs in the presence of sex hormones. IL-5 production before and after stimulation with GS-9620 (first panel), hormone treatments of estradiol (E2), progesterone (P4), and danazol (dana) alone (middle panel), or in the presence of GS-9620 (right panel). Teal symbols are female donors (A), and peach symbols are male donors (B). (C) Fold induction of IL-5 production by biological sex (left) and age (right). Mann-Whitney test and nonparametric spearman correlation were used to calculate p values.

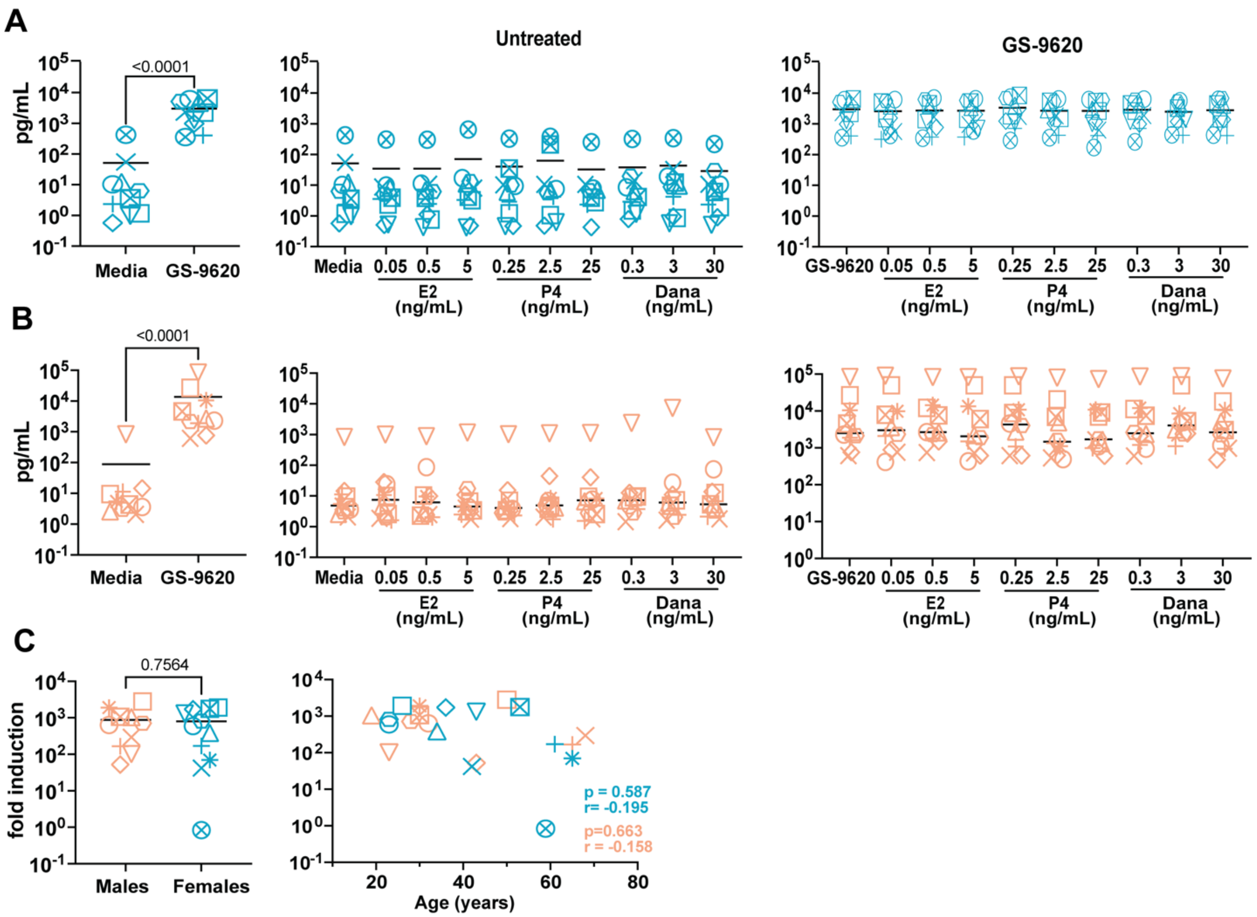

SF9. Analysis of GS-9620-mediated IL-6 induction in PBMCs in the presence of sex hormones. IL-6 production before and after stimulation with GS-9620 (first panel), hormone treatments of estradiol (E2), progesterone (P4), and danazol (dana) alone (middle panel), or in the presence of GS-9620 (right panel). Teal symbols are female donors (A), and peach symbols are male donors (B). (C) Fold induction of IL-6 production by biological sex (left) and age (right). Mann-Whitney test and nonparametric spearman correlation were used to calculate p values.

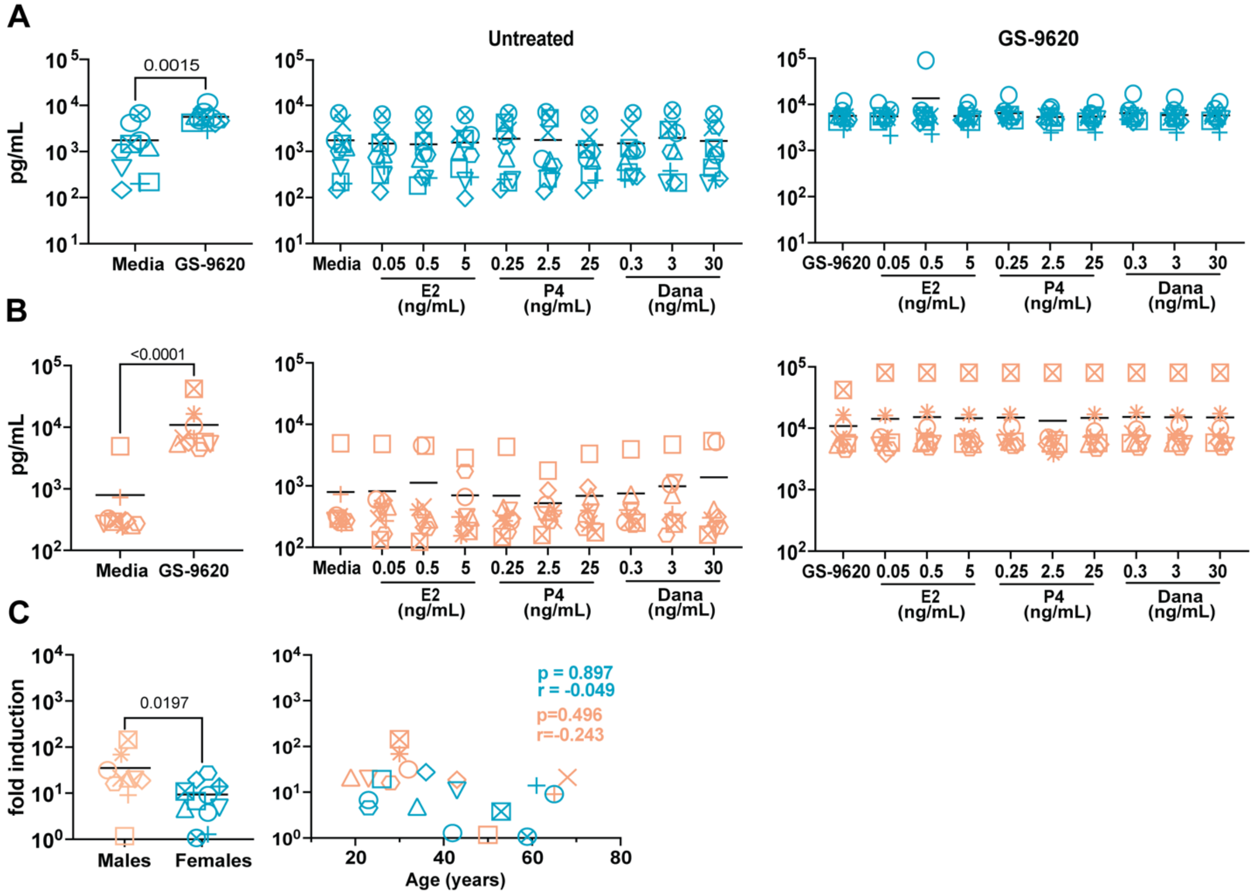

SF10. Analysis of GS-9620-mediated IL-8 induction in PBMCs in the presence of sex hormones. IL-8 production before and after stimulation with GS-9620 (first panel), hormone treatments of estradiol (E2), progesterone (P4), and danazol (dana) alone (middle panel), or in the presence of GS-9620 (right panel). Teal symbols are female donors (A), and peach symbols are male donors (B). (C) Fold induction of IL-8 production by biological sex (left) and age (right). Mann-Whitney test and nonparametric spearman correlation were used to calculate p values.

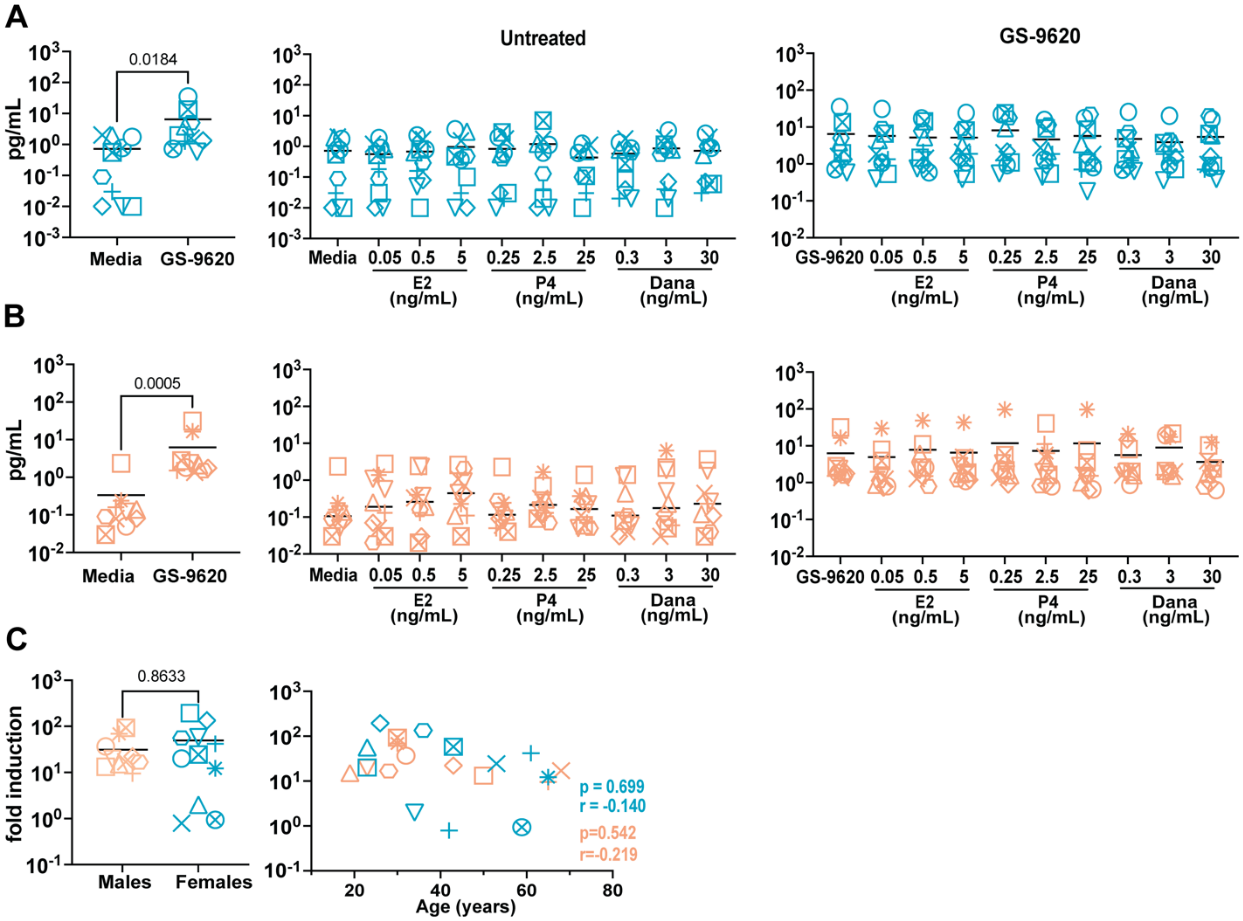

SF11. Analysis of GS-9620-mediated IL-22 induction in PBMCs in the presence of sex hormones. IL-22 production before and after stimulation with GS-9620 (first panel), hormone treatments of estradiol (E2), progesterone (P4), and danazol (dana) alone (middle panel), or in the presence of GS-9620 (right panel). Teal symbols are female donors (A), and peach symbols are male donors (B). (C) Fold induction of IL-22 production by biological sex (left) and age (right). Mann-Whitney test and nonparametric spearman correlation were used to calculate p values.

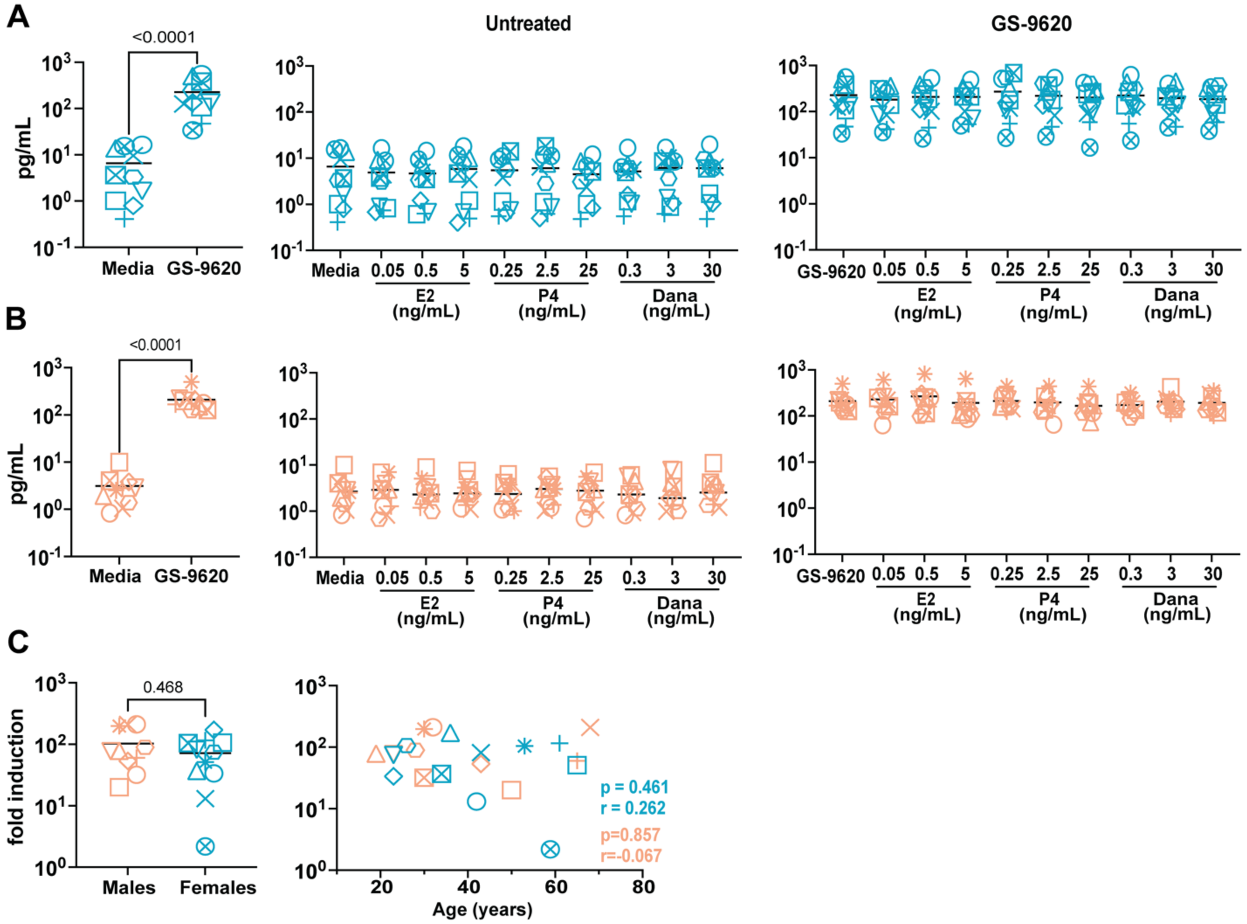

SF12. Analysis of GS-9620-mediated IL-10 induction in PBMCs in the presence of sex hormones. IL-10 production before and after stimulation with GS-9620 (first panel), hormone treatments of estradiol (E2), progesterone (P4), and danazol (dana) alone (middle panel), or in the presence of GS-9620 (right panel). Teal symbols are female donors (A), and peach symbols are male donors (B). (C) Fold induction of IL-10 production by biological sex (left) and age (right). Mann-Whitney test and nonparametric spearman correlation were used to calculate p values.

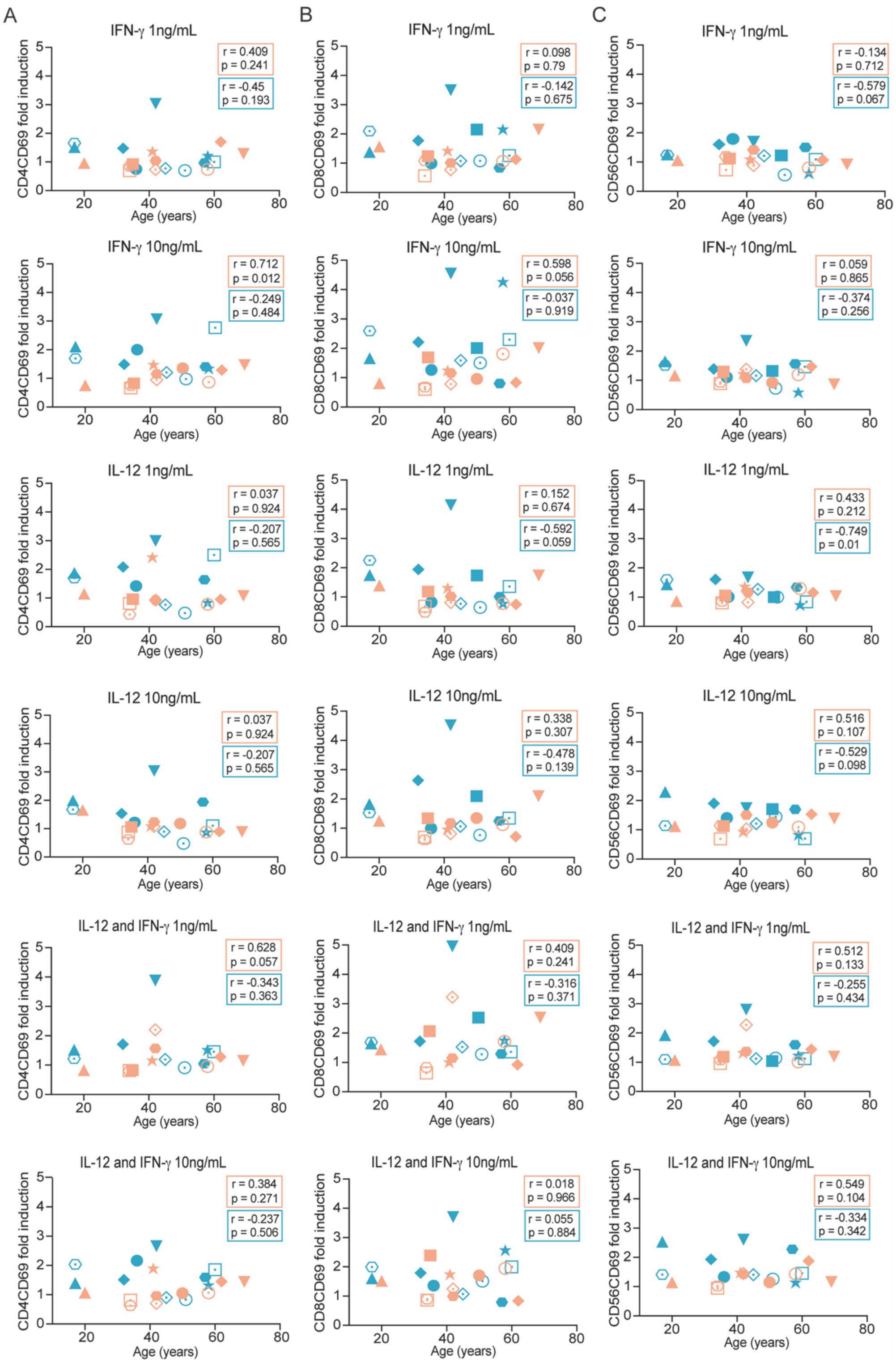

SF13. Immune cell activation treating with IFN-γ and IL-12 shows no correlation with age. Fold induction CD4 T cells (A), CD8 T cells (B), and NK cells (C) treated with IFN-γ at 1 and 10ng/mL (first and second), IL-12 at 1 and 10ng/mL (third and fourth panels), and a combination of both IFN-γ and IL-12 at 1 and 10ng/mL (fifth and sixth panels). Teal symbols are female donors, and peach symbols are male donors. Nonparametric spearman correlation was used to calculate r and p values.
